# Alpha-linolenic acid-modified liposomes associate with and modulate antibiotic activity against Helicobacter pylori

**DOI:** 10.1099/mic.0.001562

**Published:** 2025-05-30

**Authors:** Nicola C. Osborne, Rosa Catania, Snow Stolnik, Karen Robinson

**Affiliations:** 1 Nottingham Digestive Diseases Biomedical Research Centre, Biomolecular Discovery Institute, School of Medicine, University of Nottingham, Nottingham, NG7 2RD, UK; 2 Division of Molecular Therapeutics and Formulation, School of Pharmacy, University of Nottingham, Nottingham, NG7 2RD, UK

**Keywords:** antibiotics, fatty acids, *Helicobacter pylori*, linolenic acid, liposomes

## Abstract

Fatty acids have antimicrobial activity against a wide range of bacteria. We therefore aimed to incorporate omega-3 unsaturated *alpha*-linolenic acid (αLA) into the membrane of antibiotic-loaded liposomes to create a system with dual antibacterial activity against *Helicobacter pylori*. Liposomes containing 1-palmitoyl-2-oleoyl-sn-glycero-3-phosphocholine, cholesterol, sphingomyelin and the far-red fluorescent DiD label, with varying content of αLA (mol% to total lipid), were fabricated using the thin film evaporation method and hydrated with PBS or amoxicillin solution. The liposomes were characterized for αLA and amoxicillin content, particle size, membrane fluidity and permeability, prior to their addition to cultures of *H. pylori* strains and clinical isolates. αLA-modified liposomes enhanced the antibacterial action of amoxicillin against *H. pylori*, as determined using a viable count method. The liposomal formulation achieved a 3-log reduction in bacterial density, compared to a 1.5- to 2-log reduction by amoxicillin in solution. The application of imaging cytometry revealed a significantly increased association of αLA-modified liposomes with *H. pylori* cells, compared to non-αLA control liposomes. In conclusion, this study demonstrated, for the first time, that the incorporation of αLA increased the attraction of the liposomes to *H. pylori* and increased antibiotic potency. This suggests that αLA incorporation into liposomes may not only act as an antimicrobial, but also as a potential *in vivo* targeting strategy.

## Graphical Abstract

Summary of key findings: (1) Linolenic acid (αLA) was effective in killing *Helicobacter pylori*, but not *Escherichia coli* or *Campylobacter jejuni*. (2) Liposomes encapsulating αLA and amoxicillin had greatly enhanced bactericidal activity against *H. pylori*, compared to liposomes containing just one of these components, or amoxicillin in solution. (3) Liposomes encapsulating αLA rapidly bound to *H. pylori* at higher densities compared to liposomes without αLA. Created in BioRender. Robinson, K. (2025) a31l841 https://BioRender.com.

**Impact Statement**

*Helicobacter pylori* is a bacterium that commonly infects the human stomach, and it may lead to ulcers in the stomach and duodenum or gastric cancer. It is usually treated with triple therapies consisting of two antibiotics and a proton pump inhibitor to suppress stomach acid, or quadruple therapies which also contain a bactericidal bismuth compound. The options for effective antibiotics are limited to those that reach the complex niche which *H. pylori* inhabits, in the protective mucus layer of the stomach. Current antibiotic therapies are not thought to work topically in the stomach but are absorbed into the bloodstream and then must be secreted back across the stomach epithelium. Resistance to the commonly used antibiotics has been increasing over the last 20 years, and some patients are therefore given repeated rounds of therapy.

This study explored the potential benefits of formulating antibiotics with a fatty acid, *alpha*-linolenic acid, which has innate antimicrobial activity. We aimed to prepare stable liposomes, incorporating αLA into the hydrophobic lipid bilayer and with amoxicillin in the liposome lumen. We found that free αLA had antibacterial activity against multiple strains of *H. pylori*, but not against other Gram-negative bacteria (*Escherichia coli* and *Campylobacter jejuni*). Formulating both αLA and amoxicillin into 130 nm liposomes improved their antimicrobial activity, compared with soluble amoxicillin and amoxicillin liposomes formulated without αLA. We also showed that liposomes containing αLA rapidly became associated with *H. pylori*, indicating a targeting effect.

## Data Availability

The data that support the findings of this study are available from the corresponding authors upon reasonable request.

## Introduction

Fatty acids have long been known to have antimicrobial activity against a wide range of Gram-positive and Gram-negative bacteria [1,5]. They are naturally produced at many common points of entry for pathogenic microbes to the body of humans and animals, including the gastrointestinal tract, to provide innate protection from infection [6]. They have therefore been considered as possible additions to antibiotic therapy [4,5]. Fatty acids have a hydrophobic carbon chain of between less than 6 and more than 22 carbon atoms, which may contain double bonds (unsaturation), additional methyl groups from branched-chain fatty acids and a hydrophilic carboxyl group. Their antibacterial activity differs according to the length of the carbon chain and degree of unsaturation. Polyunsaturated fatty acids of between 8 and >12 carbon atoms seem to have the most prominent antibacterial activity. Gram-positive organisms appear to be more susceptible than Gram-negative bacteria [4]. Although the mechanisms remain unclear, fatty acids may be specifically taken up by bacteria. They may change the composition of the cytoplasmic membrane and disrupt normal metabolic processes such as nutrient uptake, regulation of gene expression, virulence, quorum sensing and biofilm formation [7,10]. There is growing interest in fatty acids as antimicrobials against pathogens and foodborne bacteria due to their activity without a specific antimicrobial target molecule [5,11, 12].

The most often discussed mechanism of antimicrobial activity of fatty acids against Gram-negative bacteria considers their insertion and accumulation in the outer membrane. This causes membrane destabilization, and cell lysis occurs when used at high doses [8,13]. There have been several previous reports indicating that the polyunsaturated fatty acid, C18 : 3 *alpha*-linolenic acid (αLA), acts as a potent antimicrobial against the Gram-negative human pathogen *Helicobacter pylori* [3,4, 8, 13]. One study demonstrated that when radiolabelled αLA was added to *H. pylori*, over 80% of αLA associated with the bacteria was located within the cell membrane [8]. At inhibitory concentrations, this association resulted in the loss of the bacterium’s typical spiral shape and eventually caused lysis [2,3]. These observations form the basis for a hypothesis that αLA primarily functions as a natural antimicrobial agent by disrupting *H. pylori*’s outer membrane.

*H. pylori* is a common member of the human gastric microbiota. Colonization is often established during early childhood, where it usually remains for a lifetime without causing overt symptoms [14,15]. In around 10–15% of cases, however, the infection results in peptic ulcer disease or gastric cancer. *H. pylori* is the leading cause of ulcer disease, distal gastric adenocarcinoma and gastric mucosa-associated lymphoid tissue lymphoma. Despite decades of intensive research, there is no vaccine available, and resistance to the therapeutic antibiotics is becoming more common [16,17]. *H. pylori* bacteria inhabit a niche in the thick mucus layer of the stomach, which is difficult for antibiotics to penetrate. Only a limited number of antibiotics are effective, as they do not act topically in the stomach but must be absorbed systemically and secreted across the gastric epithelial barrier into the overlying mucus layer. A triple therapy is usually given, consisting of two antibiotics (usually amoxicillin with clarithromycin or metronidazole) with a proton pump inhibitor to suppress gastric acid production [16]. The current therapies expose the microbiome of the body to antibiotics, leading to common side effects and poor patient compliance. When treatments fail to eradicate the infection, patients are given further rounds of antibiotics, which increases the risk of acquisition of resistance by other bacteria in their microbiome [18,19].

*H. pylori* has been shown to be susceptible to unsaturated fatty acids, especially linolenic acid, and spontaneous development of resistance was reported to be low [3,13]. A human clinical study investigated the effect of polyunsaturated fatty acids as a dietary supplement on *H. pylori* colonization *in vivo* but found no effect on colonization densities [20]. Potential reasons for these observations may include poor solubility within the stomach, and esterification, oxidation and reaction of the fatty acid with proteins and other food-derived compounds in the stomach. These factors could render the fatty acids inactive and also lead to precipitation, rendering them unable to diffuse into the gastric mucus and reach the *H. pylori* niche [8,21]. Treatment of *H. pylori*-infected mice with αLA also had no significant impact on *H. pylori* colonization [22]. Incorporation of αLA into drug delivery systems has hence been investigated, including in liposomal formulations, which were found to be highly bactericidal *in vitro* [23,24].

Thamphiwatana *et al.* [22] moved this work forward significantly, demonstrating that αLA loaded into liposomes caused a much greater decrease in *H. pylori* colonization in infected mice, compared to the free fatty acid, and it was significantly more efficient than conventional *H. pylori* triple therapy. Our study aimed to build on this work with the design of liposomes incorporating αLA and amoxicillin to potentially achieve a dual mechanism of action. Free αLA had good bactericidal activity against *H. pylori* strains and clinical isolates *in vitro*, but no effect was detected on *Campylobacter jejuni* or *Escherichia coli*, which are also Gram-negative bacteria. Liposome formulations containing αLA had more antibacterial activity against * H. pylori* than those containing amoxicillin in the absence of αLA. Liposomes containing both antimicrobial compounds induced the strongest antibacterial effects, and the formulations were stable.

## Methods

### Bacterial strains, isolates and culture

*H. pylori* Sydney Strain 1 (SS1) and NCTC 11637 were utilized, as previously described [25]. Clinical isolates 194A, 451A and 456A originated from patients attending a routine upper gastrointestinal tract endoscopy clinic at the Queen’s Medical Centre, Nottingham, with informed written patient consent and ethics approval. The clinical isolates were characterized for antibiotic susceptibility using E-tests in a previous study, and all were found to be sensitive to amoxicillin [26]. Isolate 194A was resistant to metronidazole and clarithromycin, and isolate 451A was resistant to metronidazole, whereas isolate 456A was sensitive to these antibiotics. The GFP-expressing *H. pylori* mutant, AB31:pSB13, was a kind gift from Dr Rob Delahay (University of Nottingham). *H. pylori* and *C. jejuni* strain NCTC 13256 were maintained on blood agar base#2 plates containing 5% horse blood (Oxoid, UK) and incubated in a MACS VA500 microaerobic workstation (Don Whitley Scientific, UK) at 37 °C in a humidified atmosphere of 86% nitrogen, 6% oxygen, 3% hydrogen and 5% carbon dioxide. *E. coli* strain B834 (Merck Life Science UK Limited) was grown aerobically on Lysogeny Broth (LB) agar plates (Oxoid) at 37 °C.

Liquid cultures of *H. pylori* and *C. jejuni* were produced by inoculating 10 ml of Brain Heart Infusion (BHI) broth (Oxoid), supplemented with 5% FCS (Merck Life Science UK Limited), and incubating overnight at 37 °C with moderate shaking under microaerophilic conditions. *E. coli* cultures were grown from a single colony in 10 ml of LB broth and incubated overnight at 37 °C with moderate shaking. For all strains, overnight cultures were centrifuged at 1,700 ***g*** for 5 min and resuspended in 2 ml of fresh medium ready for use.

### Preparation and characterization of αLA liposomes

Liposomes of varying content of αLA (mol% to total lipid) were fabricated using the classical thin film evaporation method [27]. Formulations of 1-palmitoyl-2-oleoyl-sn-glycero-3-phosphocholine (POPC; NOF Europe), cholesterol (Chol; Merck Life Science UK Limited), sphingomyelin (SM; NOF Europe) and the far-red fluorescent, lipophilic carbocyanine label [1,1′-dioctadecyl-3,3,3′,3′-tetramethylindodicarbocyanine, 4-chlorobenzenesulfonate salt (DiD)] (Thermo Fisher) were prepared in a ratio of POPC:Chol:SM:DiD at 45.25 : 40.00 : 14.55 : 0.20 mol%, without αLA (denoted as Lipo). Liposomes were also prepared with incorporated αLA at 10, 20 and 30 mol%, where an increase in αLA content was compensated by POPC (denoted as Lipo αLA_10_, Lipo αLA_20_ or Lipo αLA_30_). To prevent issues arising from αLA oxidation [28], the samples were aliquoted and stored at low temperatures, and all processes were protected from light [29]. The lipid components, POPC, Chol and DiD were dissolved in chloroform (Thermo Fisher). αLA and SM were dissolved in a 1 : 1 chloroform:methanol solvent mixture. The final total concentration of lipid components was 20 mM. The required aliquots of lipid solutions were then mixed, the organic solvent was evaporated and the lipid film was formed and dried overnight in a desiccator and further under nitrogen for 15 min. The lipid film was then hydrated with either 1 ml of PBS or in 1 ml of 5 mg ml^−1^ amoxicillin sodium salt (Cambridge Bioscience) in PBS, to prepare amoxicillin-loaded liposomes (denoted as Lipo Amx or Lipo αLA-Amx, depending on the absence or presence of αLA, respectively).

The mixture was sonicated for 10 min in an ultrasonic bath and then extruded through a polycarbonate membrane with 0.1 µm pore size (Whatman Nucleopore), using an extruder (Avanti Polar Lipids) to produce 1.0 ml of each liposome formulation. Separation of unincorporated (free) amoxicillin from liposomes was carried out by size exclusion chromatography using a PD-10 column (GE Healthcare) with PBS as the elution buffer, according to the manufacturer’s protocol.

DiD fluorescence emission prior to, and following, removal of free drugs by chromatography was used to assess the total lipid concentration of final samples (Tecan Infinite 200 PRO multimode plate reader, with excitation and emission wavelengths of 610 and 670 nm, respectively). The hydrodynamic particle size of fabricated liposomes was determined by dynamic light scattering (DLS) using a Malvern Nano-ZS Zetasizer (Malvern, UK). Measurements were repeated three times for each sample.

### Quantification of linolenic acid in liposomes

Two hundred microlitres of samples of liposome suspensions were freeze-dried and dissolved in 550 µl of DMSO-d_6_ (Merck Life Science UK Limited). Fifty microlitres of a 10 mg ml^−1^ benzoic acid stock (Merck Life Science UK Limited) were added as the internal standard. αLA was quantified by ^1^H NMR on a Bruker 400MHz NMR spectrometer using the following equation, where *C* is the molar concentration and DF is the dilution factor of 3. The NMR spectra were analysed, and peaks were integrated using MestReNova analytical chemistry software.


$$C_{\alpha LA}=\left(\frac{{Integral}_{\alpha LA}}{{Integral}_{Benzoicacid}}\right)\times \left(\frac{{No.ofProtons}_{Benzoicacid}}{{No.ofProtons}_{\alpha LA}}\right)\times C_{Benzoicacid}\times DF$$

### Quantification of amoxicillin in liposomes

The concentration of amoxicillin loading into the liposomes was quantified using a ninhydrin colourimetric assay. Thirty milligrams of hydrindantin and 200 mg of ninhydrin (Merck Life Science UK Limited) were dissolved in 7.5 ml of DMSO and protected from light. Immediately prior to analysis, 2.5 ml of 4 M sodium acetate was added. Twofold serial dilutions of amoxicillin sodium in PBS, from 500 to 1.95 µg ml^−1^, were used as standards, with PBS as a negative control. Liposomes without amoxicillin were used as blanks. One hundred microlitres of standards or liposomes were added in triplicate to 96-well plates. Seventy-five microlitres of ninhydrin solution were added, and the plate was placed in a water bath at 80 °C, protected from light, for 30 min. One hundred microlitres of 50% isopropanol (Thermo Fisher) were then added to all wells, and the absorbance was read on a Tecan plate reader (as above) at 570 nm.

### Laurdan probe assessment of liposome membrane fluidity

Liposomes containing 0, 10, 20 and 30 mol% αLA were prepared with the addition to the lipid film of 0.2 mol% 6-dodecanoyl-2-dimethylaminonaphthalene (Laurdan; Thermo Fisher), compensated by POPC. Laurdan-containing liposome suspensions, diluted 1 : 5 in PBS, were added in triplicate to 96-well plates and incubated for 1 h at either 4, 16, 37 or 45 °C before reading on a Tecan microplate reader (as above) at the same temperature. Laurdan fluorescence was read at an excitation wavelength of 350 nm and emission wavelengths of 440 and 490 nm. The generalized polarization (GP) of Laurdan was calculated as below, where I_440_ is the fluorescence intensity at 440 nm and I_490_ is the fluorescence intensity at 490 nm:


$$GP=\frac{I_{440}-I_{490}}{I_{440}+I_{490}}$$

The scale of GP values ranges from −1 to +1, where a high GP value indicates low membrane fluidity [30,31].

### Evaluation of liposome permeability using carboxyfluorescein

Carboxyfluorescein (CF; Merck Life Science UK Limited) was used to quantify the leakage of water-soluble compounds from the aqueous core of the liposomes. Dry lipid films containing 0, 10, 20 or 30% αLA were prepared and hydrated with 1 ml of 50 mM CF in PBS. Unincorporated CF was removed using a PD-10 column, and the liposome particle size was measured. Liposomes were diluted 1 : 5 in PBS and 1 : 5 in 0.2% Triton X-100 in triplicate in 96-well plates. The plate was incubated for 1 h at either 4, 15, 37 or 45 °C, and fluorescence was read at excitation and emission wavelengths of 485 and 518 nm, respectively, on a microplate reader (Tecan, as above). The percentage CF release was determined by the following equation:


$$\%\text{CF release}=\left(\frac{\text{Mean fluorescence of liposome in PBS}}{\text{Mean fluorescence of liposome in 0.2\textbackslash{}\% Triton X-100 buffer}}\right)\times 100$$

### Bacterial growth curves

Miles and Misra viable count assays [32] were used to determine the growth of *H. pylori*, *C. jejuni* and *E. coli*, under various treatment conditions. Overnight cultures of *H. pylori* and *C. jejuni* were produced by inoculating 10 ml of BHI broth (Oxoid), supplemented with 5% FCS (Merck Life Science UK Limited), and incubating at 37 °C with moderate shaking under microaerophilic conditions. *E. coli* cultures were grown in 10 ml of LB broth and incubated overnight at 37 °C with moderate shaking. For all strains, the overnight cultures were centrifuged at 1,700 ***g*** for 5 min and resuspended in 2 ml of fresh medium at a starting OD of 0.05 at a wavelength of 600 nm (OD_600_). This equates to ~5×10^6^ c.f.u. ml^−1^.

The suspensions were added to triplicate wells of a sterile 96-well plate (NUNC), with free αLA or amoxicillin, or liposome formulations, to a total well volume of 200 µl. Plates were incubated with shaking at 37 °C, with *H. pylori* under microaerophilic conditions for up to 24 h, prior to plating 10 µl of tenfold serial dilutions onto blood agar plates (*H. pylori* or *C. jejuni*) or LB agar plates (*E. coli*). * H. pylori* plates were incubated at 37 °C under microaerophilic conditions for 3–5 days, prior to colony counting and calculation of c.f.u. per millilitre in the culture wells; *C. jejuni* plates were incubated for 2 days, and *E. coli* plates were incubated for 24 h. The minimal bactericidal concentration (MBC) was calculated as that reduces the c.f.u. per millilitre by 99.9%, compared to the c.f.u. per millilitre in control wells containing no antimicrobial compound or formulation.

#### Imaging cytometry

A total of 40 µg ml^−1^ liposomes containing the fluorescent DiD dye and incorporating αLA (Lipo αLA), and the equivalent concentration of control liposomes without αLA (Lipo), were incubated with the AB31:pSB13 GFP-expressing *H. pylori* strain, at an OD_600_ of 0.05, in BHI broth with 5% FCS to a total volume of 200 µl in 96-well plates. Plates were incubated for 1 min and 10 min at 37 °C under microaerophilic conditions, with moderate shaking. Equivalent concentrations of GFP-positive bacteria alone were used as negative controls. After incubation, the samples were centrifuged, washed with PBS, fixed in 4% paraformaldehyde for 15 min and resuspended in PBS before undergoing analysis on an Amnis^®^ ImageStream^®^ cytometer. The fixation was applied as we were not permitted to use live bacteria in the cytometer. Data on 10,000 events were acquired from a live gate of GFP-positive *H. pylori* events.

All events were first plotted as area against aspect ratio and gated to include single bacterial events. Only bacterial images that were in focus were gated on and included in the analysis. Of these focused bacterial populations, the events were analysed according to the fluorescent intensity of GFP and DiD signals. The proportions of GFP-positive events that were DiD-positive and -negative were calculated.

## Results

### αLA antibacterial activity

**Fig. 1.**
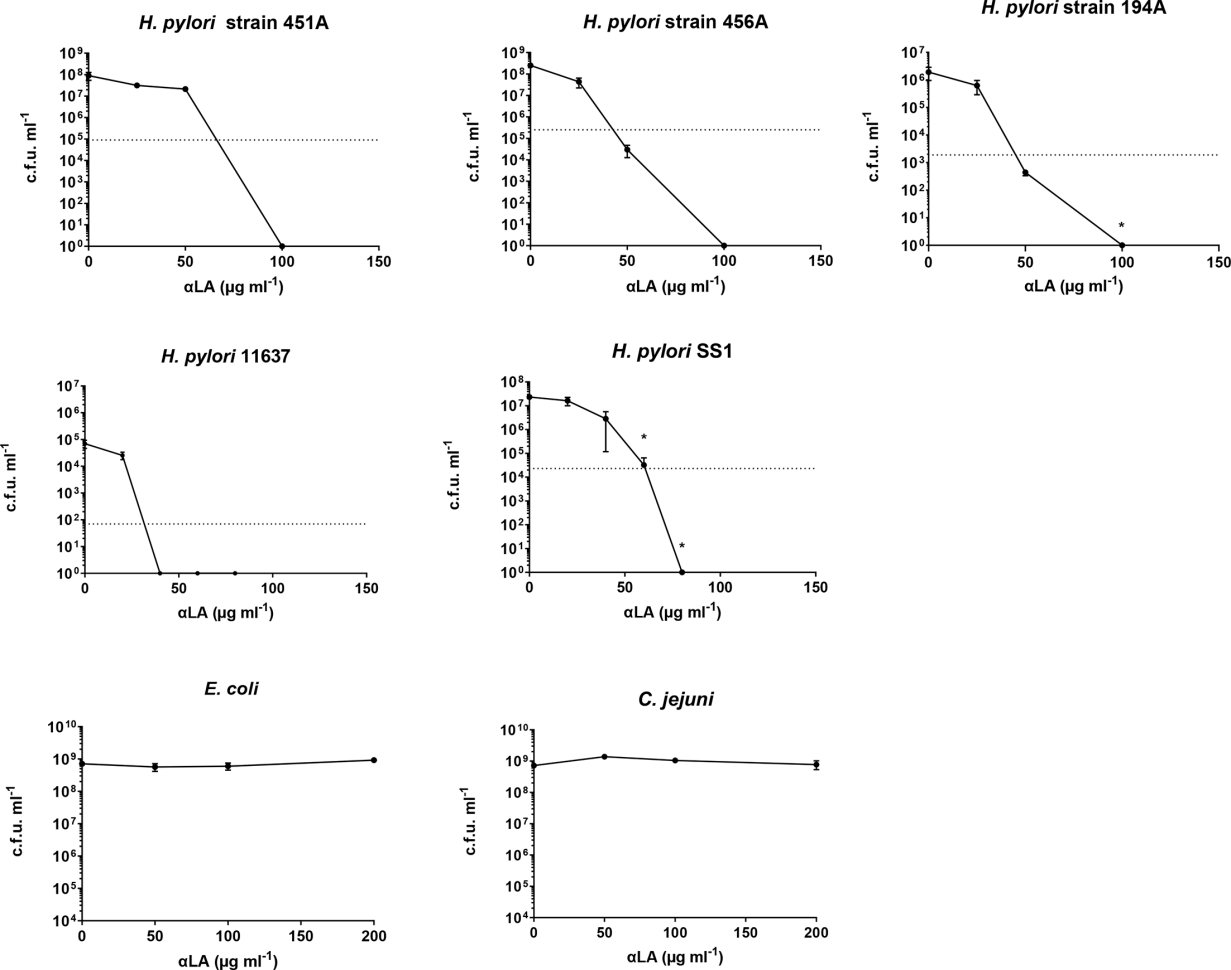
The *in vitro* antimicrobial activity of ‘free’ linolenic acid (αLA) against *H. pylori* clinical isolates, 451A, 456A and 194A; laboratory strains 11637 and SS1; and *E. coli* B8341 and *C. jejuni* NCTC 13256 strains. c.f.u. per millilitre was calculated from viable counts after 24 h of incubation. Means of three replicates from three independent experiments were plotted (*N*=3, *n*=3), error bars=sem. In these experiments, αLA was initially dissolved in DMSO, and this solution dispersed in the relevant culture medium (final DMSO concentrations of less than 2% v/v). The dotted line represents 99.9% of c.f.u. per millilitre from control cultures with 0 µg ml^−1^ αLA. *P<0.05 compared to 0 µg ml^−1^ αLA.

The antibacterial activity of ‘free’ αLA was tested against three clinical isolates (including two known to be antibiotic-resistant) and three laboratory strains of *H. pylori*, and two other Gram-negative bacterial strains: *E. coli* and *C. jejuni* (Fig. 1). There was a marked reduction in c.f.u. per millilitre of all of the *H. pylori* strains after 24 h of incubation with αLA. No colonies of the *H. pylori* SS1 and 11637 strains were recovered following culture with 80 µg ml^−1^ and 40 µg ml^−1^ αLA, respectively. The MBCs were calculated to be 61 µg ml^−1^ and 31 µg ml^−1^ αLA for strains SS1 and 11637, respectively, and 66 µg ml^−1^, 42 µg ml^−1^ and 45 µg ml^−1^ αLA for the clinical isolates 451A, 456A and 194A. Surprisingly, there was no detectable reduction in c.f.u. per millilitre of *E. coli* or *C. jejuni* when incubated with up to 200 µg ml^−1^ αLA.

### Production and analysis of liposomes with incorporated linolenic acid (LipoαLA)

**Fig. 2.**
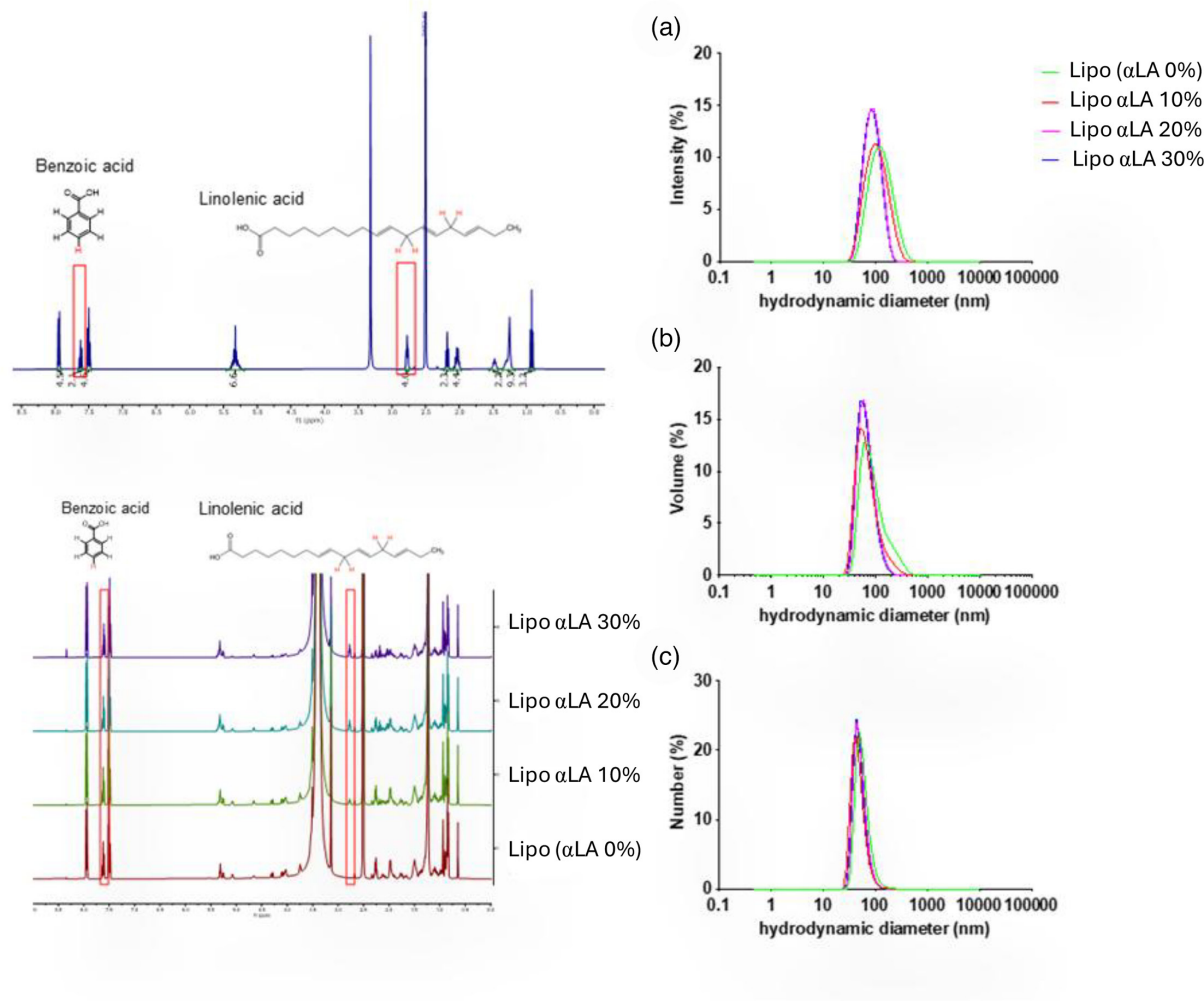
Representative ^1^H NMR spectra of free αLA and αLA-containing liposomes (Lipo αLA). Freeze-dried liposome samples and free αLA were dissolved in DMSO-d_6_ with 500 µg of benzoic acid as the internal standard. Red boxes highlight the benzoic acid and αLA representative peaks, at 7.6 and 2.78 ppm, respectively, integrated for the quantification of αLA. % refers to mol% of αLA as a proportion of the total lipid composition. Characterization of αLA-containing liposomes by DLS. Particle size distribution by intensity (**a**), volume (**b**) and number (**c**).All measurements repeated three times at room temperature. αLA % refers to the mol% of αLA in relation to the total lipid composition of the liposomes.

^1^H-NMR analysis of αLA incorporation into liposomes prepared at 10, 20 and 30 αLA mol% (Fig. 2) revealed incorporation efficacies of 89, 86 and 96 %, respectively. This resulted in αLA concentrations of 442, 684 and 1,097 µg ml^−1^ in liposome suspensions, respectively. Particle size analysis by DLS of liposomes containing 0, 10, 20 or 30 mol% αLA was presented as intensity, volume and particle size distributions (Fig. 2). The profiles indicated relatively narrow particle size distributions of liposomes with the desired average sizes in the range of 50–200 nm for all formulations.

### Effect of αLA incorporation on liposomal membrane fluidity and permeability

**Fig. 3.**
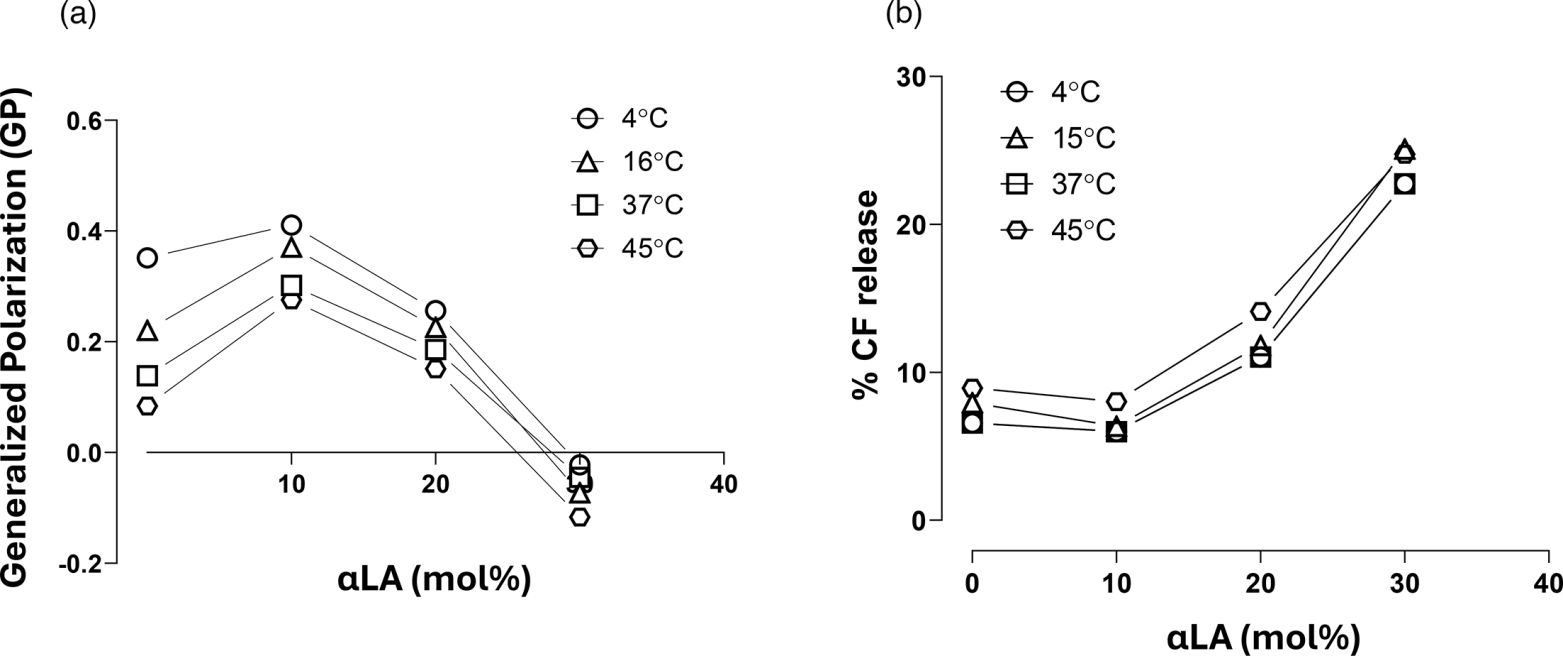
The effect of linolenic acid (αLA) on the GP of liposome membranes after 1 h incubation at 4 °C, 16 °C, 37 °C and 45 °C. Each condition was tested in triplicate (**a**). The effect of linolenic acid (αLA) on the liposome permeability to CF measured after 1 h incubation at 4 °C, 15 °C, 37 °C and 45 °C, in triplicate (**b**). mol% refers to the molar % of αLA amongst the total liposome lipid concentration.

We prepared liposomes with and without αLA, incorporating the fluorescent Laurdan dye to measure membrane fluidity via a GP analysis. The emitted fluorescence spectrum of Laurdan is affected by its environment in the membrane. Less densely packed (less ordered) lipid membranes tend to have increased water penetration. Proximity of the dye to water results in a shift in the fluorescence emission spectrum, which correlates with membrane fluidity and can be assessed using the Laurdan GP equation [30,31, 33]. On incorporation of 10 mol% αLA, the GP values increased compared to those without αLA, indicating less fluidity and that the membrane order of the liposomes increased (Fig. 3). As the composition of αLA in the liposomes was increased from 10 to 30 mol%, however, a decrease in the GP values was noted, indicating a progressively less ordered structure. GP data on the liposomes showed a complex relationship of αLA content with incubation temperature. There was a clear trend of decreasing GP values for ‘control’ liposomes (without αLA) as incubation temperatures increased, indicating a decrease in membrane order with increased temperature. The temperature effect on GP was gradually diminished as the αLA content increased to 20 mol% and above. At 30 mol% αLA content, liposomes showed low GP values for all tested temperatures, indicating that higher αLA incorporation had a dramatic effect on membrane fluidity (disorder), even at temperatures below room temperature.

To balance the effect of αLA on lipid packing, SM (15 mol%) and a relatively high content of Chol (40 mol%) were included in the liposome formulation, as per their reported effect on increasing lipid packing in a bilayer [34,35].

The change in membrane permeability at different temperatures and αLA content was also assessed by the release of an encapsulated water-soluble CF fluorescent probe from the liposomes (Fig. 3). CF self-quenches at high concentrations, such as those inside the liposomes, and upon leakage out of the liposome, where concentrations are lower, it fluoresces. CF was encapsulated into liposomes at a saturation concentration of 0.2 mM.

The permeability of the liposomal membrane was most pronounced at 30 mol% αLA content, for all temperatures tested. This may be the result of a disordered (fluid) lipid bilayer for all formulations of 30 mol% αLA, as indicated by the Laurdan GP values. At 20 mol% αLA incorporation, the CF release at the 1 h time point was about 10%, which was considered reasonable.

### Comparison of the antibacterial activity of amoxicillin in solution and encapsulated into αLA liposomes

**Fig. 4.**
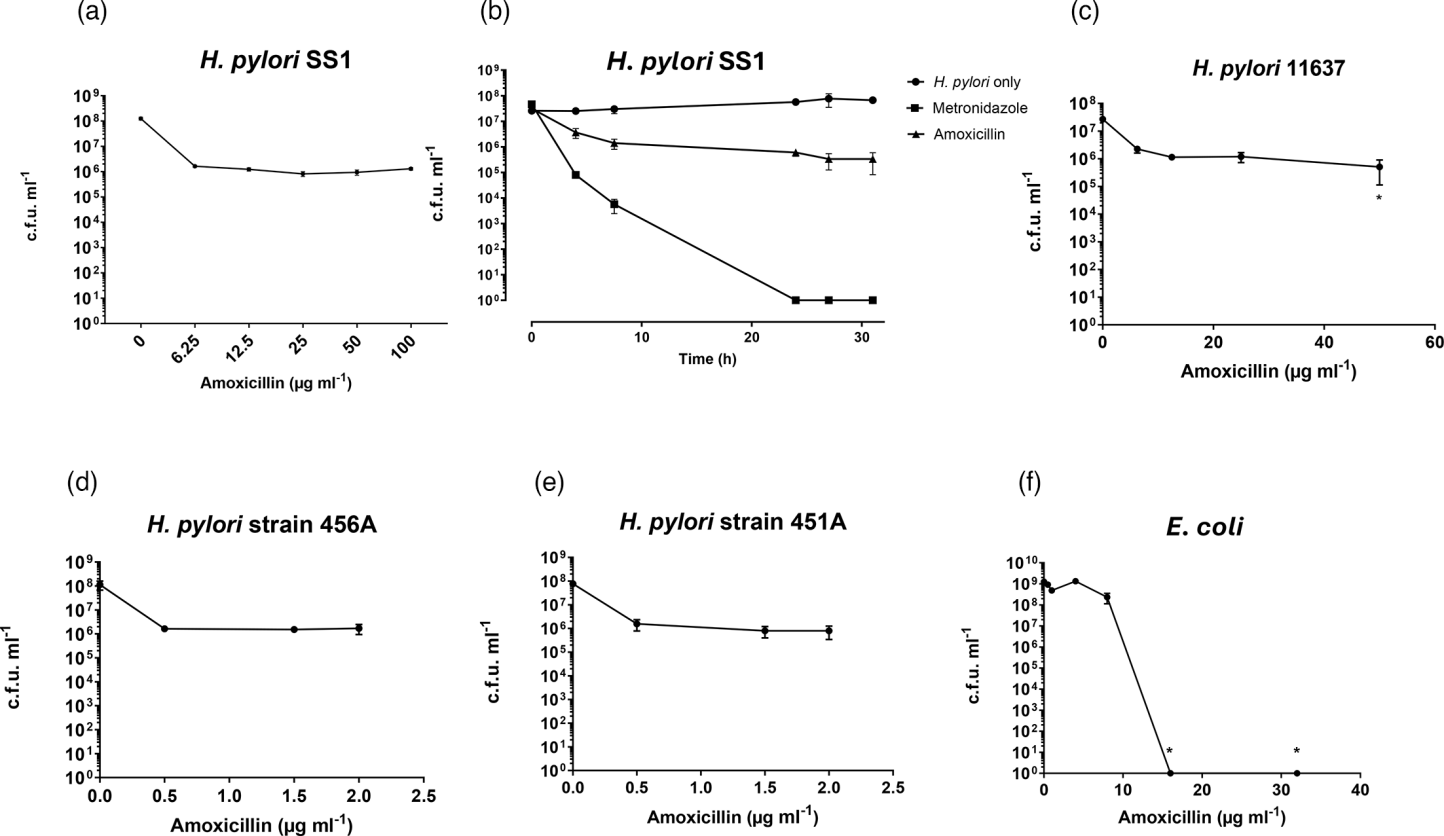
The *in vitro* antimicrobial activity of ‘free’ amoxicillin in solution to bacteria in broth culture. (a) Amoxicillin activity against *H. pylori* SS1 strain; (**b**) *H. pylori* SS1 strain growth over 31 h. c.f.u. per millilitre is shown at 0, 4, 7.5, 24, 27 and 31 h incubation with 10 µg ml^−1^ of amoxicillin or 10 µg ml^−1^ metronidazole; (**c**) activity of amoxicillin against *H. pylori* 11637 strain; (**d,** e) amoxicillin activity against *H. pylori* 456A and 451A clinical isolates; (**f**) amoxicillin activity against *E. coli* B834 strain. Unless otherwise stated, bacteria were incubated for 24 h with the indicated concentrations of amoxicillin. The mean c.f.u. per millilitre of three replicates is shown from three independent experiments plotted (*N*=3, *n*=3); error bars are sem. *P<0.05 compared to untreated.

The activity of ‘free’ amoxicillin in solution on broth cultures of *H. pylori* laboratory strains and clinical isolates was assessed (Fig. 4) prior to examining its activity in liposomal formulations. All of the strains were susceptible to amoxicillin when exposed in E-test strip assays on agar plates (MICs of <0.125 µg ml^−1^) [26]. The broth cultures containing concentrations of up to 100 µg ml^−1^ resulted in ~1.5- to 2-log decreases in *H. pylori* c.f.u. per millilitre after an initial 8 h of exposure to the antibiotic, followed by a stabilized c.f.u. per millilitre during a further 24 h of incubation. The MBC, defined as the concentration required to kill 99.9 % of bacteria, i.e. a 3-log reduction in c.f.u. per millilitre, was calculated to be 10 µg ml^−1^. None of the *H. pylori* strains tested were completely killed (to 0 c.f.u. ml^−1^) at the applied concentrations of amoxicillin; however, 10 µg ml^−1^ metronidazole included as an assay control was completely effective. Our tests of the antibacterial effects of amoxicillin against *E. coli* in broth cultures showed complete killing of the bacteria.

Amoxicillin was encapsulated into the liposome formulation of 20 mol% of αLA (Lipo αLA_20_-Amx). The selection of αLA_20_ content was based on the liposomal membrane properties and permeability data (Fig. 3). The encapsulation efficiency of amoxicillin was low, at just 1.8% in liposomes without αLA (LipoAmx) and at 2.5 % in Lipo αLA_20_-Amx formulations containing 20 mol% αLA. The particle size and size distribution of Lipo αLA_20_-Amx liposomes did not significantly change upon loading of liposomes with amoxicillin (Table 1).

**Table 1.** Characteristics of liposomes, following encapsulation of amoxicillin

Formulation	Amoxicillin loading (µg ml^−1^)	Encapsulation efficiency (%)	Particle size (nm)
LipoAmx	57.3±16.8	1.80±0.1	138.9±27.7
Lipo αLA_20_-Amx	85.1±16.6	2.55±0.9	108.7±15.9

A 5 mg ml−1 amoxicillin sodium solution was used for the liposome hydration step. Data are represented as the mean±sd from *n*=3. There was no significant difference in the loading of amoxicillin when αLA was present in the liposomes.

The antibacterial activity of the amoxicillin-loaded liposomes against *H. pylori* SS1 was tested using the previously described broth culture method. Liposomes with incorporated αLA (Lipo αLA), liposomes loaded with amoxicillin (LipoAmx) and liposomes with incorporated αLA (20 mol%) and loaded with amoxicillin (Lipo αLA_20_Amox) at different loading levels were compared.

**Fig. 5.**
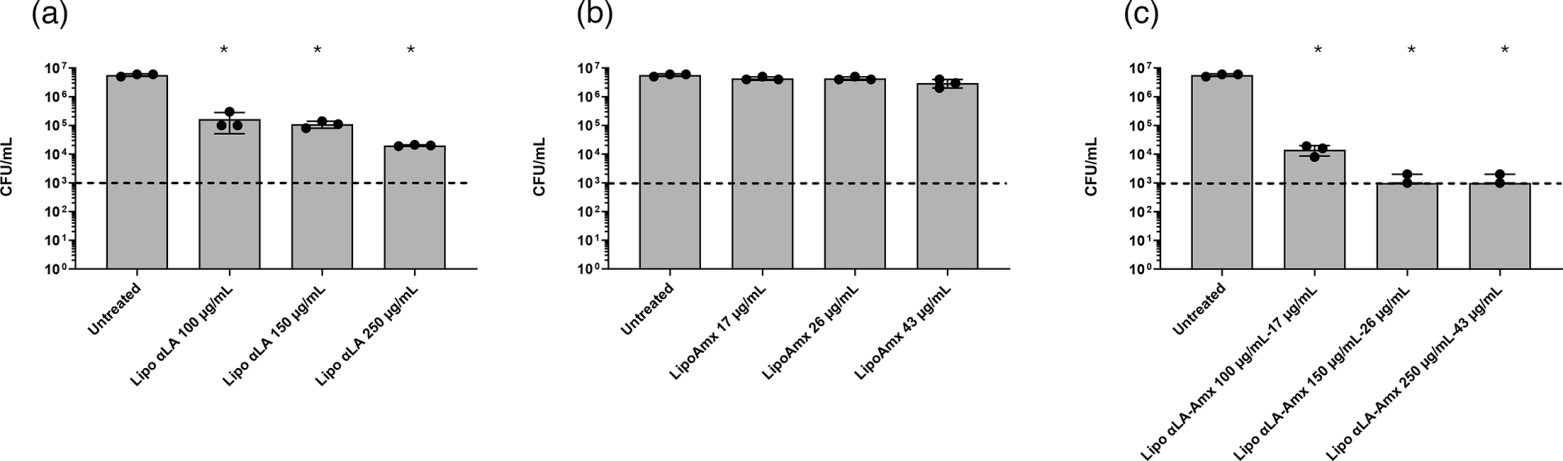
Antimicrobial activity against *H. pylori* SS1, expressed as c.f.u. per millilitre. Data are presented for liposomes with incorporated αLA (Lipo αLA) (**a**), amoxicillin-loaded liposomes (LipoAmx) (**b**) and liposomes with incorporated αLA (20 mol%) and loaded with amoxicillin (Lipo αLA-Amx) (**c**) at different applied concentrations. Bacteria were incubated with liposomes for 24 h, and the means of triplicate replicates from three independent experiments with the three formulations were plotted (*N*=3, *n*=3). Error bars=sd, *P<0.05 (Friedman test with Dunn’s multiple comparison test, compared to untreated bacteria). Formulations were arranged into low, medium and high doses: Lipo αLA=100, 150 and 250 µg ml^−1^ of αLA; LipoAmx=17, 26 and 43 µg ml^−1^ of amoxicillin; Lipo αLA-Amx=100 µg ml^−1^ αLA and 17 µg ml^−1^ amoxicillin, 150 µg ml^−1^ αLA and 26 µg ml^−1^ amoxicillin or 250 µg ml^−1^ αLA and 43 µg ml^−1^ amoxicillin. The dotted line represents the MBC, a 3-log decrease in c.f.u. per millilitre.

Incorporation of αLA into liposomes significantly reduced its antimicrobial action, compared to that observed in its free form. A total of 80 µg ml^−1^ free αLA yielded 100% killing of the SS1 strain from 2×10^7^ c.f.u. ml^−1^ in the untreated control (shown in Fig. 1). Following incubation with liposomes containing 100 µg ml^−1^ or 250 µg ml^−1^ αLA (Lipo αLA), 1.7×10^5^ c.f.u. ml^−1^ and 2.0×10^4^ c.f.u. ml^−1^ remained, respectively, compared with 5.7×10^6^ c.f.u. ml^−1^ in the untreated control (Fig. 5). The liposomes loaded with amoxicillin (LipoAmx) had no significant antibacterial activity at concentrations of 17 µg ml^−1^ (4.3×10^6^ c.f.u. ml^−1^) and 43 µg ml^−1^ (3.0×10^6^ c.f.u. ml^−1^), even though a 100-fold decrease in c.f.u. per millilitre had been noted with as little as 6 µg ml^−1^ free amoxicillin in solution (Fig. 4). Formulating both materials into the liposomes reversed these effects. Liposomes incorporating αLA and loaded with amoxicillin (Lipo αLA_20_Amox) achieved 3-log decreases in c.f.u. per millilitre at two different concentrations applied: 150 µg ml^−1^ αLA and 26 µg ml^−1^ amoxicillin and 250 µg ml^−1^ αLA and 43 µg ml^−1^ amoxicillin.

The data indicate that the encapsulation of amoxicillin within liposomes inhibited its ability to directly interact with the bacterial surface and traverse into bacterial cells. When amoxicillin was encapsulated within αLA-containing liposomes, this appeared to have enhanced their ability to kill the bacteria. To see if the presence of αLA in the liposomes changed their interaction with the cell surface, we therefore performed imaging flow cytometry experiments and compared interactions between the fabricated liposomal formulations and *H. pylori*.

### Imaging cytometry analysis of liposome and bacterial interactions

**Fig. 6.**
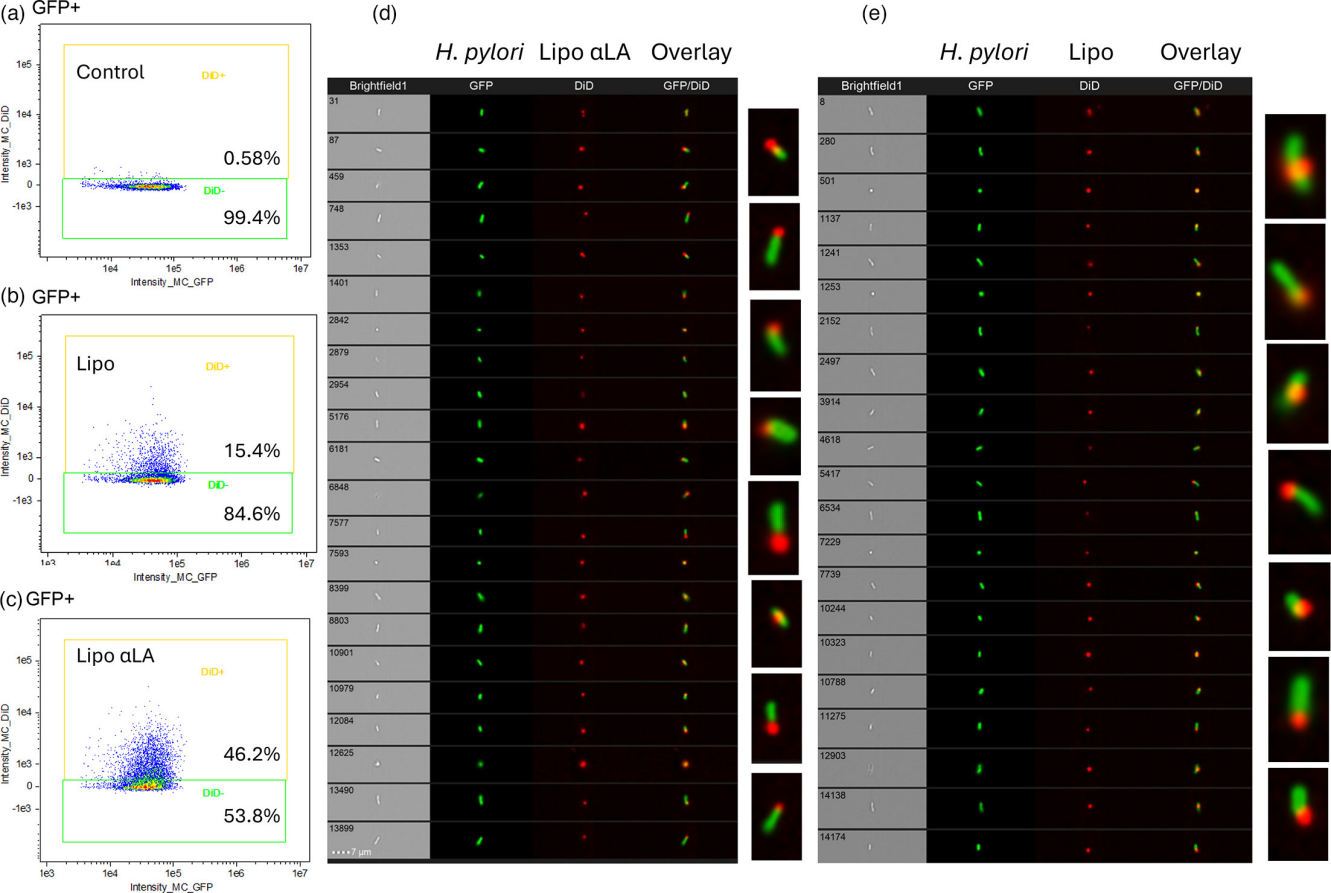
Flow cytometry plots from incubation of GFP-expressing *H. pylori* AB31 with PBS (**a**), liposomes without αLA (Lipo) (**b**) or liposomes with incorporated linolenic acid (Lipo αLA) (**c**). The liposomes were labelled with DiD red fluorescent dye. Gating for DiD^+^ and DiD^−^ populations within the GFP^+^ population is shown for each combination (**a–c**). Panels d and e show representative images of individual GFP^+^/DiD^+^ events taken on the Amnis ImageStream^®^ flow cytometer after 10 min incubation of GFP-expressing *H. pylori* AB31 with DiD-labelled αLA-containing liposomes (Lipo αLA) (**d**) or DiD-labelled liposomes without linolenic acid (Lipo) (**e**), respectively. Green=GFP, red=DiD.

In order to investigate differences in the bacterial interactions of liposome formulations with and without αLA, imaging cytometry was employed to visualize a GFP-expressing strain of *H. pylori* (AB31:pSB13, green fluorescence) with DiD-labelled liposomes (red fluorescence). Liposomes with and without αLA (20 mol%) were compared. Data from focused images of 10,000 GFP^+^ cells were acquired. The data were analysed according to the percentages of DiD^+^ and DiD^−^ events (cells associated with, and not associated with DiD-labelled liposomes), and the median intensity of fluorescence (MFI). Fig. 6 presents imaging cytometry data as population dot plots, with representative images of individual cells. Table 2 displays the percentages of DiD^+^ cells and the MFI.

**Table 2.** Image cytometry data following incubation of GFP-expressing *H. pylori* with DiD-labelled liposomes for 1 and 10 min

GFP-*H. pylori* treatment and incubation time	GFP^+^	DiD^+^ of GFP^+^ events	DiD^−^ of GFP^+^ events
**DiD-labelledLipo αLA, 1** **min**	100% (6,247 events)	41.8%MFI of DiD=922.84	58.2%MFI of DiD=83.72
**DiD-labelledLipo, 1** **min**	100% (6,466 events)	9.93%MFI of DiD=777.86	90.1%MFI of DiD=16.99
**PBS, 1** **min**	100% (6,406 events)	0.3%MFI of DiD=510.15	99.7%MFI of DiD=0.67
**DiD-labelledLipo αLA, 10** **min**	100% (5,983 events)	46.2%MFI of DiD=954.52	53.8%MFI of DiD=85.91
**DiD-labelledLipo, 10** **min**	100% (6,214 events)	15.4%MFI of DiD=831.94	84.6%MFI of DiD=23.33
**PBS, 10** **min**	100% (6,253 events)	0.58%MFI of DiD=511.41	99.4%MFI of DiD=4.46

Interactions of GFP+ *H. pylori* with DiD-labelled liposome formulations, with 20 mol% linolenic acid (Lipo αLA) and without (Lipo), were compared. *H. pylori* treated with PBS (no liposomes) was included as a control. A live gate was set for 10,000 GFP+ events corresponding to *H. pylori* cells. From this, the population of focused singlet cells (the 100% GFP+ gate) was analysed for the frequencies of DiD+ and DiD− events, and the median DiD fluorescent intensities (MFI) in these groups.

After a very short period of incubation, followed by washing, higher proportions of DiD^+^ events and a corresponding higher DiD MFI were obtained from bacteria treated with Lipo αLA liposomes, indicating a higher density of Lipo αLA liposomes bound to the cells compared to the liposomes without αLA (Table 2, Fig. 6). A total of 41.8% of the GFP^+^ *H. pylori* cells became DiD-positive (DiD^+^) via exposure to the Lipo αLA for 1 min (Table 2). This was compared with only 9.93% of DiD^+^ cells when liposomes without αLA were applied. A similar result was observed after 10 min of incubation. Some low-intensity red fluorescence was present in the *H. pylori* treated with PBS as a control (Table 2, Fig. 6a), likely to be autofluorescence. The images indicate an association between the liposomes and *H. pylori* which, interestingly, appeared to occur preferentially at the poles of the bacterial cells.

## Discussion

Initial experiments showed a marked reduction in c.f.u. per millilitre after 24 h of incubation with αLA for all of the *H. pylori* strains tested. Similar findings have been reported in previous studies [3,13, 23] and attributed to αLA-mediated disruption and destabilization of the bacterial membrane. Despite the potent antimicrobial activity against *H. pylori*, αLA had no effect on Gram-negative organisms *E. coli* and *C. jejuni*. The data are in agreement with previous reports showing a lack of antimicrobial activity of αLA, and other related unsaturated fatty acids, against *E. coli* [36,37] and other Gram-negative bacteria [21,37, 38].

The incorporation of αLA into liposomes was found to be of high efficiency, with an average particle size of 139 nm. This size was desirable to balance a need for a diffusion of liposomes through the gastric mucus where *H. pylori* resides [39] with a reasonable drug loading. There was no indication of species in the micellar size range in the side distribution profiles (usually <10 nm), which would be expected if unincorporated αLA self-assembled into small vesicles or micelles in a medium of neutral pH [40].

We found an increase in membrane order of liposomes upon the addition of 10 mol% of αLA, which was unexpected. It has been suggested that unsaturated fatty acids may leave space for the planar Chol to fill [41]. It is possible that at above 10 mol% αLA, the disruption to packing by αLA could not be consolidated by Chol [42]. The permeability of the liposomal membrane was most pronounced at 30 mol% αLA, as expected from the Laurdan GP values, indicating disordered lipid bilayers. A similar increase in liposome permeability has also been reported for other liposomes which incorporated polyunsaturated fatty acids [42,43].

In general, several factors could affect the lipid packing of the bilayer membrane, including temperature, Chol content, fatty acid chain length and unsaturated lipid content [35,41, 44,47]. Looser packing of unsaturated chains due to ‘kinks’ caused by double bonds has been reported to lead to disordered membrane structure in the liquid crystalline phase at lower temperatures [48,51]. We found that incorporation of αLA, with its three double bonds, into liposome membranes caused increased membrane fluidity, and the effects were also temperaturedependent. The presence of Chol and SM in lipid bilayers has been reported to increase the lipid packing [34,35], and Chol has been demonstrated to increase the resistance of liposomal membranes to changes in temperature [52,55].

All of the *H. pylori* strains in the study were susceptible to amoxicillin when exposed in E-test strip assays on agar plates (MICs of <0.125 µg ml^−1^ [26]), but results from the broth cultures of *H. pylori* with amoxicillin showed a much smaller effect than expected. This could not simply be ascribed to amoxicillin resistance but may be due to differences in the protocols. In the broth microdilution method, the bacteria were removed from the broth containing the antibiotic and plated onto antibiotic-free agar for colony counting. In E-tests, however, the antibiotic is present both during growth and evaluation of the zone of inhibition. The inability of amoxicillin to completely kill *H. pylori* in broth cultures *in vitro* has also been reported by other groups, where 1×10^6^ c.f.u. ml^−1^ remained after 24 h of incubation [56,57]. It has been indicated that a prolonged incubation of 2–3 days may be needed for complete *H. pylori* killing in liquid cultures [58]. The outer membrane of Gram-negative bacteria contains porins that act as barriers to some beta-lactam antibiotics, but amoxicillin’s highly hydrophilic properties allow it to readily diffuse through these channels which provides a therapeutic advantage [59,60].

The liposomal encapsulation efficiency of amoxicillin was low, which agrees with published studies on hydrophilic drugs [61,62]. Despite low amoxicillin loading, one could argue that the therapeutic dose required would be lower relative to systemic application [61], if the encapsulated antibiotic reaches the *H. pylori* gastric niche and preferentially interacts with bacterial cells [63].

Incorporation of either αLA or amoxicillin into the liposomes dramatically reduced their antimicrobial action, when compared to their free forms. This could be expected, as the formulation prevents direct interactions of compounds with bacterial cells until their release occurs. Conversely, formulating αLA and amoxicillin together into liposomes reversed this. Using techniques including transmission electron microscopy (EM) and scanning EM, Jung *et al.* [24] were previously able to show that liposomes containing αLA caused a breakdown in the integrity and stability of the *H. pylori* outer membrane, resulting in leakage of cytoplasmic contents and cell lysis. This occurred rapidly, within 5 min. In *H. pylori*-infected mice, treatment with liposomal αLA reduced inflammation and increased bacterial clearance compared to the standard triple therapy. The group also demonstrated that free and liposomal αLA could successfully kill *H. pylori* in its coccoid form [22]. In our study, Lipo αLA liposomes did not yield similar bacteria-killing results in the absence of amoxicillin. The difference may possibly be in the liposome lipid composition; the liposomes used by Jung *et al.* comprised l-α-phosphatidylcholine, Chol and αLA in a 6 : 1 : 3 wt ratio [24], whereas liposomes in our study contained POPC, Chol, SM and DiD at 45 : 40 : 14.5 : 0.2 with 10, 20 or 30% αLA (compensated by POPC). This composition aimed to balance liposomal membrane fluidity, arising from αLA, with their capacity to retain and deliver encapsulated water-soluble antibiotic. The presence of Chol and SM contributes to the organization of lipid packing in membranes. It is hence likely that the liposomes in this study have different, potentially lower, membrane fluidity to those in the Jung *et al.* study. Differing compositions of the αLA liposomes could potentially affect their ability to interact with the bacterial surface. Indeed, Wang *et al.* showed that the addition of Chol to liposomes reduced their membrane fluidity and markedly decreased their ability to fuse with the Gram-negative bacterium *Pseudomonas aeruginosa* [64].

The data provided by imaging flow cytometry showed that the incorporation of αLA into the liposomal membrane significantly increased its association with *H. pylori* cells. Interestingly, the association appeared to occur preferentially at the bacterial poles. Further studies are required to investigate the nature of these interactions. The Lipo αLA liposomes appeared to be more fusogenic than liposomes without αLA, although they were found to have similar fluidity at 37 °C to αLA-free liposomes, as determined by GP. The surface charge of the liposomes was not determined in this work; however, αLA-containing liposomes can be expected to have a more negative surface charge than αLA-free liposomes, as previously reported in the literature [24]. *H. pylori* also has a negative surface charge; therefore, the increased attraction of αLA liposomes is unlikely to be related to charge [65]. *H. pylori*’s outer membrane has a unique fatty acid profile [66], including the presence of linoleic acid, a fatty acid similar to linolenic acid but with one less double bond in the acyl chain [67]. It is possible that *H. pylori* could have a higher affinity to membranes which have a higher fatty acid profile. Chol is essential for *H. pylori* growth, as it is unable to synthesize it. The organism actively scavenges exogenous Chol and is able to detect and migrate towards Chol in the environment [68]. Although liposomes with and without αLA had equivalent Chol loading, this may be another mechanism which promotes bacterial interaction with the liposomes. The enhanced bacterial killing by cell-associated liposomes likely resulted in fusion of the liposomes with the bacterial surface at the points of ‘contact’, and thereby more efficient release of amoxicillin encapsulated in the liposomal aqueous core to the bacteria. The liposomes appeared to frequently bind to * H. pylori* at the poles of the bacteria. Further work is needed to understand why this may be, and additionally, we plan to test whether the liposomes formulated with αLA can overcome resistance in amoxicillin-resistant isolates, with studies both *in vitro* and *in vivo.*

## Conclusion

We prepared liposomes encapsulating amoxicillin and αLA and found them to be capable of killing *H. pylori in vitro*. The study demonstrated that the presence of αLA increased the affinity of the liposomes to *H. pylori*. This suggests that αLA not only acts as an antimicrobial but also as a potential targeting factor for *H. pylori*, which could be used to further develop antibiotic formulations for more efficient eradication of the infection.

## Abbreviations

BHI: Brain Heart Infusion
CF: carboxyfluorescein
Chol: cholesterol
DiD: 1,1′-dioctadecyl-3,3,3′,3′-tetramethylindodicarbocyanine, 4-chlorobenzenesulfonate salt
DLS: dynamic light scattering
GP: generalized polarization
LB: Lysogeny Broth
MBC: minimal bactericidal concentration
MFI: median intensity of fluorescence
POPC: 1-palmitoyl-2-oleoyl-sn-glycero-3-phosphocholine
SM: sphingomyelin
SS1: Sydney Strain 1
αLA: *alpha*-linolenic acid

## References

[1] Kabara JJ, Swieczkowski DM, Conley AJ, Truant JP (1972). Fatty acids and derivatives as antimicrobial agents. Antimicrob Agents Chemother.

[2] Khulusi S, Ahmed HA, Patel P, Mendall MA, Northfield TC (1995). The effects of unsaturated fatty acids on *Helicobacter* pylori in vitro. J Med Microbiol.

[3] Thompson L, Cockayne A, Spiller RC (1994). Inhibitory effect of polyunsaturated fatty acids on the growth of *Helicobacter* pylori: a possible explanation of the effect of diet on peptic ulceration. Gut.

[4] Yoon BK, Jackman JA, Valle-González ER, Cho N-J (2018). Antibacterial free fatty acids and monoglycerides: biological activities, experimental testing, and therapeutic applications. Int J Mol Sci.

[5] Borreby C, Lillebæk EMS, Kallipolitis BH (2023). Anti-infective activities of long-chain fatty acids against foodborne pathogens. FEMS Microbiol Rev.

[6] Fischer CL (2020). Antimicrobial activity of host-derived lipids. Antibiotics.

[7] Fischer CL, Drake DR, Dawson DV, Blanchette DR, Brogden KA (2012). Antibacterial activity of sphingoid bases and fatty acids against gram-positive and gram-negative bacteria. Antimicrob Agents Chemother.

[8] Jung SW, Lee SW (2016). The antibacterial effect of fatty acids on *Helicobacter pylori* infection. Korean J Intern Med.

[9] Kumar P, Lee JH, Beyenal H, Lee J (2020). Fatty acids as antibiofilm and antivirulence agents. Trends Microbiol.

[10] Lowden MJ, Skorupski K, Pellegrini M, Chiorazzo MG, Taylor RK (2010). Structure of vibrio cholerae toxt reveals a mechanism for fatty acid regulation of virulence genes. Proc Natl Acad Sci USA.

[11] Park S, Lee JH, Kim YG, Hu L, Lee J (2022). Fatty acids as aminoglycoside antibiotic adjuvants against *Staphylococcus aureus*. Front Microbiol.

[12] Baker LY, Hobby CR, Siv AW, Bible WC, Glennon MS (2018). *Pseudomonas aeruginosa* responds to exogenous polyunsaturated fatty acids (PUFAs) by modifying phospholipid composition, membrane permeability, and phenotypes associated with virulence. BMC Microbiol.

[13] Sun CQ, O’Connor CJ, Roberton AM (2003). Antibacterial actions of fatty acids and monoglycerides against *Helicobacter pylori*. FEMS Immunol Med Microbiol.

[14] Robinson K, Atherton JC (2021). The spectrum of helicobacter-mediated diseases. Annu Rev Pathol.

[15] White JR, Winter JA, Robinson K (2015). Differential inflammatory response to *Helicobacter pylori* infection: etiology and clinical outcomes. J Inflamm Res.

[16] Malfertheiner P, Megraud F, Rokkas T, Gisbert JP, Liou J-M (2022). Management of *Helicobacter pylori* infection: the maastricht VI/Florence consensus report. Gut.

[17] Katelaris P, Hunt R, Bazzoli F, Cohen H, Fock KM (2023). *Helicobacter pylori* world gastroenterology organization global guideline. J Clin Gastroenterol.

[18] Wang L, Yao H, Tong T, Lau K, Leung SY (2022). Dynamic changes in antibiotic resistance genes and gut microbiota after *Helicobacter pylori* eradication therapies. Helicobacter.

[19] Sjomina O, Vangravs R, Ļeonova E, Poļaka I, Pūpola D (2024). Clarithromycin-containing triple therapy for *Helicobacter pylori* eradication is inducing increased long-term resistant bacteria communities in the gut. Gut.

[20] Duggan AE, Atherton JC, Cockayne A, Balsitis M, Evison S (1997). Clarification of the link between polyunsaturated fatty acids and *Helicobacter pylori*-associated duodenal ulcer disease: a dietary intervention study. Br J Nutr.

[21] Desbois AP, Smith VJ (2010). Antibacterial free fatty acids: activities, mechanisms of action and biotechnological potential. Appl Microbiol Biotechnol.

[22] Thamphiwatana S, Gao W, Obonyo M, Zhang L (2014). In vivo treatment of *Helicobacter pylori* infection with liposomal linolenic acid reduces colonization and ameliorates inflammation. Proc Natl Acad Sci USA.

[23] Obonyo M, Zhang L, Thamphiwatana S, Pornpattananangkul D, Fu V (2012). Antibacterial activities of liposomal linolenic acids against antibiotic-resistant *Helicobacter pylori*. Mol Pharm.

[24] Jung SW, Thamphiwatana S, Zhang L, Obonyo M (2015). Mechanism of antibacterial activity of liposomal linolenic acid against *Helicobacter pylori*. PLoS One.

[25] Winter JA, Letley DP, Cook KW, Rhead JL, Zaitoun AA (2014). A role for the vacuolating cytotoxin, VacA, in colonization and *Helicobacter pylori*-induced metaplasia in the stomach. J Infect Dis.

[26] Garvey E, Rhead J, Suffian S, Whiley D, Mahmood F (2023). High incidence of antibiotic resistance amongst isolates of *Helicobacter pylori* collected in Nottingham, UK, between 2001 and 2018. J Med Microbiol.

[27] Bangham AD, De Gier J, Greville GD (1967). Osmotic properties and water permeability of phospholipid liquid crystals. Chem Phys Lipids.

[28] Wu GS, Stein RA, Mead JF (1982). Autoxidation of phosphatidylcholine liposomes. Lipids.

[29] Grit M, Crommelin DJ (1993). Chemical stability of liposomes: implications for their physical stability. Chem Phys Lipids.

[30] Gaus K, Zech T, Harder T (2006). Visualizing membrane microdomains by Laurdan 2-photon microscopy. Mol Membr Biol.

[31] Sengupta S, Karsalia R, Morrissey A, Bamezai AK (2021). Cholesterol-dependent plasma membrane order (Lo) is critical for antigen-specific clonal expansion of CD4+ T cells. Sci Rep.

[32] Miles AA, Misra SS, Irwin JO (1938). The estimation of the bactericidal power of the blood. J Hyg.

[33] Parasassi T, De Stasio G, Ravagnan G, Rusch RM, Gratton E (1991). Quantitation of lipid phases in phospholipid vesicles by the generalized polarization of laurdan fluorescence. Biophys J.

[34] Semple SC, Leone R, Wang J, Leng EC, Klimuk SK (2005). Optimization and characterization of a sphingomyelin/cholesterol liposome formulation of vinorelbine with promising antitumor activity. J Pharm Sci.

[35] Slotte JP (1999). Sphingomyelin-cholesterol interactions in biological and model membranes. Chem Phys Lipids.

[36] Dilika F, Bremner PD, Meyer JJM (2000). Antibacterial activity of linoleic and oleic acids isolated from *Helichrysum pedunculatum*: a plant used during circumcision rites. Fitoterapia.

[37] Zheng CJ, Yoo J-S, Lee T-G, Cho H-Y, Kim Y-H (2005). Fatty acid synthesis is a target for antibacterial activity of unsaturated fatty acids. FEBS Lett.

[38] Bergsson G, Steingrímsson O, Thormar H (2002). Bactericidal effects of fatty acids and monoglycerides on *Helicobacter pylori*. Int J Antimicrob Agents.

[39] Norris DA, Puri N, Sinko PJ (1998). The effect of physical barriers and properties on the oral absorption of particulates. Adv Drug Deliv Rev.

[40] Wang Y, Jiang L, Shen Q, Shen J, Han Y (2017). Investigation on the self-assembled behaviors of C18 unsaturated fatty acids in arginine aqueous solution. RSC Adv.

[41] Sułkowski WW, Pentak D, Nowak K, Sułkowska A (2005). The influence of temperature, cholesterol content and pH on liposome stability. J Mol Struct.

[42] Muranushi N, Takagi N, Muranishi S, Sezaki H (1981). Effect of fatty acids and monoglycerides on permeability of lipid bilayer. Chem Phys Lipids.

[43] Rasti B, Jinap S, Mozafari MR, Yazid AM (2012). Comparative study of the oxidative and physical stability of liposomal and nanoliposomal polyunsaturated fatty acids prepared with conventional and Mozafari methods. Food Chem.

[44] Aguilar LF, Pino JA, Soto-Arriaza MA, Cuevas FJ, Sánchez S (2012). Differential dynamic and structural behavior of lipid-cholesterol domains in model membranes. PLoS One.

[45] de Meyer F, Smit B (2009). Effect of cholesterol on the structure of a phospholipid bilayer. Proc Natl Acad Sci USA.

[46] Kaddah S, Khreich N, Kaddah F, Charcosset C, Greige-Gerges H (2018). Cholesterol modulates the liposome membrane fluidity and permeability for a hydrophilic molecule. Food Chem Toxicol.

[47] Nyholm TKM, Engberg O, Hautala V, Tsuchikawa H, Lin K-L (2019). Impact of acyl chain mismatch on the formation and properties of sphingomyelin-cholesterol domains. Biophysical J.

[48] Seu KJ, Cambrea LR, Everly RM, Hovis JS (2006). Influence of lipid chemistry on membrane fluidity: tail and headgroup interactions. Biophys J.

[49] Small DM (1984). Lateral chain packing in lipids and membranes. J Lipid Res.

[50] Edidin M (2003). The state of lipid rafts: from model membranes to cells. Annu Rev Biophys Biomol Struct.

[51] Epand RM, Epand RF, Ahmed N, Chen R (1991). Promotion of hexagonal phase formation and lipid mixing by fatty acids with varying degrees of unsaturation. Chem Phys Lipids.

[52] Ferreira TM, Coreta-Gomes F, Ollila OHS, Moreno MJ, Vaz WLC (2013). Cholesterol and POPC segmental order parameters in lipid membranes: solid state 1H–13C NMR and MD simulation studies. Phys Chem Chem Phys.

[53] Parasassi T, Di Stefano M, Loiero M, Ravagnan G, Gratton E (1994). Influence of cholesterol on phospholipid bilayers phase domains as detected by laurdan fluorescence. Biophys J.

[54] Stott BM, Vu MP, McLemore CO, Lund MS, Gibbons E (2008). Use of fluorescence to determine the effects of cholesterol on lipid behavior in sphingomyelin liposomes and erythrocyte membranes. J Lipid Res.

[55] van Blitterswijk WJ, van der Meer BW, Hilkmann H (1987). Quantitative contributions of cholesterol and the individual classes of phospholipids and their degree of fatty acyl (un)saturation to membrane fluidity measured by fluorescence polarization. Biochemistry.

[56] Makobongo MO, Einck L, Peek RM, Merrell DS (2013). In vitro characterization of the anti-bacterial activity of SQ109 against *Helicobacter pylori*. PLoS One.

[57] Villegas I, Rosillo MÁ, Alarcón-de-la-Lastra C, Vázquez-Román V, Llorente M (2021). Amoxicillin and clarithromycin mucoadhesive delivery system for *Helicobacter pylori* infection in a mouse model: characterization, pharmacokinetics, and efficacy. Pharmaceutics.

[58] Dore MP, Osato MS, Realdi G, Mura I, Graham DY (1999). Amoxycillin tolerance in *Helicobacter pylori*. J Antimicrob Chemother.

[59] Kim SW, Lee JS, Park SB, Lee AR, Jung JW (2020). The Importance of porins and β-Lactamase in outer membrane Vesicles on the hydrolysis of β-lactam antibiotics. IJMS.

[60] Ghai I (2023). A barrier to entry: examining the bacterial outer membrane and antibiotic resistance. Appl Sci.

[61] Akbarzadeh A, Rezaei-Sadabady R, Davaran S, Joo SW, Zarghami N (2013). Liposome: classification, preparation, and applications. Nanoscale Res Lett.

[62] Nii T, Ishii F (2005). Encapsulation efficiency of water-soluble and insoluble drugs in liposomes prepared by the microencapsulation vesicle method. Int J Pharm.

[63] Daraee H, Etemadi A, Kouhi M, Alimirzalu S, Akbarzadeh A (2016). Application of liposomes in medicine and drug delivery. Artif Cells Nanomed Biotechnol.

[64] Wang Z, Ma Y, Khalil H, Wang R, Lu T (2016). Fusion between fluid liposomes and intact bacteria: study of driving parameters and in vitro bactericidal efficacy. Int J Nanomedicine.

[65] Westmeier D, Posselt G, Hahlbrock A, Bartfeld S, Vallet C (2018). Nanoparticle binding attenuates the pathobiology of gastric cancer-associated *Helicobacter pylori*. Nanoscale.

[66] Scherer C, Müller K-D, Rath P-M, Ansorg RAM (2003). Influence of culture conditions on the fatty acid profiles of laboratory-adapted and freshly isolated strains of *Helicobacter pylori*. J Clin Microbiol.

[67] O’Toole PW, Clyne M (2001). Helicobacter Pylori: Physiology and Genetics:.

[68] Wunder C, Churin Y, Winau F, Warnecke D, Vieth M (2006). Cholesterol glucosylation promotes immune evasion by *Helicobacter pylori*. Nat Med.

